# Chromatographic Behaviour Predicts the Ability of Potential Nootropics to Permeate the Blood-Brain Barrier

**DOI:** 10.3797/scipharm.1208-19

**Published:** 2012-10-14

**Authors:** Oldřich Farsa

**Affiliations:** Institute of Chemical Drugs, Faculty of Pharmacy, University of Veterinary and Pharmaceutical Sciences Brno, 612 42, Brno, Czech Republic.

**Keywords:** Hansch analysis, Blood-brain barrier, RP-HPLC, Embedded amide moiety, Nootropics

## Abstract

The log BB parameter is the logarithm of the ratio of a compound’s equilibrium concentrations in the brain tissue versus the blood plasma. This parameter is a useful descriptor in assessing the ability of a compound to permeate the blood-brain barrier. The aim of this study was to develop a Hansch-type linear regression QSAR model that correlates the parameter log BB and the retention time of drugs and other organic compounds on a reversed-phase HPLC containing an embedded amide moiety. The retention time was expressed by the capacity factor log k′. The second aim was to estimate the brain’s absorption of 2-(azacycloalkyl)acetamidophenoxyacetic acids, which are analogues of piracetam, nefiracetam, and meclofenoxate. Notably, these acids may be novel nootropics. Two simple regression models that relate log BB and log k′ were developed from an assay performed using a reversed-phase HPLC that contained an embedded amide moiety. Both the quadratic and linear models yielded statistical parameters comparable to previously published models of log BB dependence on various structural characteristics. The models predict that four members of the substituted phenoxyacetic acid series have a strong chance of permeating the barrier and being absorbed in the brain. The results of this study show that a reversed-phase HPLC system containing an embedded amide moiety is a functional *in vitro* surrogate of the blood-brain barrier. These results suggest that racetam-type nootropic drugs containing a carboxylic moiety could be more poorly absorbed than analogues devoid of the carboxyl group, especially if the compounds penetrate the barrier by a simple diffusion mechanism.

## Introduction

The blood-brain barrier (BBB) is one of the most important barriers in the human body. It protects brain and spinal cord tissues against unwanted compounds. Endothelial cells and brain capillaries comprise the barrier. Unlike other capillaries in the body, spaces between capillaries in the BBB are filled and tightened by tight junctions, which are membrane formations that are produced by parenchymal cells from the brain. Although the BBB can be considered both a physical barrier and a complex biochemical interface with important physiological functions, such as specific uptake by the brain [1], several transport mechanisms are involved in allowing drugs and other xenobiotics to pass through the BBB. These mechanisms are absorptive-mediated transcytosis, receptor-mediated transcytosis, passive transport, and carrier-mediated transport [2]. Most drugs are transported into the brain by means of passive transport or simple diffusion. It is generally accepted that the transport velocity depends on the lipophilicity of the compound. When the lipophilicity is expressed as log P (octanol/water), the optimal values generally range between 1.5 and 2.7, and the maximum value of log P is typically around 2.1 [1]. However, an increasing body of evidence suggests that the velocity of passive transport also depends on hydrogen bonding. The log BB parameter, which is the logarithm of the equilibrium brain/blood concentrations ratio, is frequently used to assess the likelihood that a compound can permeate the BBB. The parameter is expressed as follows: log BB = c_brain_/c_blood_. Young and co-workers realised that log BB values of a small set of six centrally acting histamine H_2_ antagonists correlated much more closely to the difference Δlog P, where Δlog P = log P (octanol/water) – log P (cyclohexane/water), than with log P (octanol/water) itself [3]. These results were in accordance with the results of Seiler, who had previously introduced the parameter Δlog P as a measure of the overall hydrogen-bonding capacity of a compound [4]. The logarithm of the capacity factor log k′ is expressed in equation 1

**Fig. 1:**
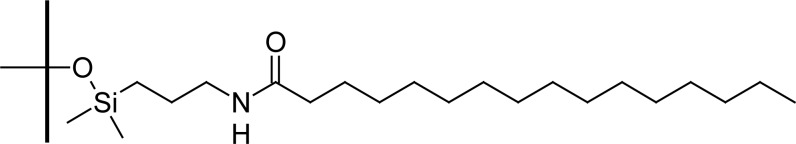
The structure of a reversed-phase column system containing an “embedded” amide moiety (such as Supelcosil ABZ Plus+ used in this study).

Eq. 1. $$\text{log}\;{\text{k}}^{\prime }=\text{log}\;[({\text{t}}_{\text{r}}-{\text{t}}_{0})/{\text{t}}_{\text{r}}],$$

where t_r_ is the retention time of the compound, and t_0_ is the dead retention time, namely the retention time of a compound that is not retained on the column. Log k′ is a measure of the position of a sample peak during column chromatography [either gas chromatography (GC) or high-performance liquid chromatography (HPLC)]. If log k′ is calculated from the retention time of a compound in a reversed-phase high-performance liquid chromate-graphy (RP-HPLC) system, it can be used as a substitute for a log P value. The partition between the hydrophobic stationary phase and the hydrophilic mobile phase is the primary means by which compounds separate on a column. Notably, log k′ values depend on the environment in which the constituent parameters are measured. Octadecyl silica gel phases represent a purely lipophilic environment, whereas a reversed-phase system containing an “embedded” amide moiety (Figure 1) may be able to account for the hydrogen bonding that may occur during the partitioning process.

The aim—and simultaneously the novelty—of this study was to mimic the behaviour of drugs and other organic compounds at the BBB by using RP-HPLC embedded with an amide moiety. Specifically, the aim was to find a function (equation 2)

**Fig. 2:**
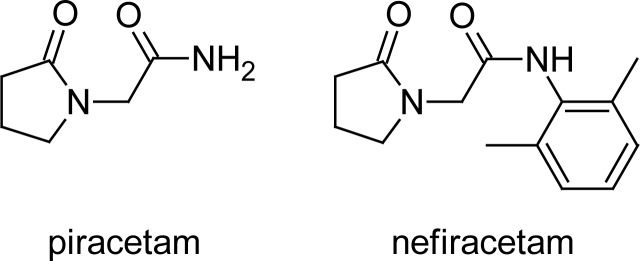
Structures of piracetam and nefiracetam.

Eq. 2. $$\text{log}\;\text{BB}=\text{f }(\text{log}\;{\text{k}}^{\prime })$$

by means of regression analysis and to subsequently use it to predict whether some potential and experimental nootropic drugs that may function as novel CNS therapeutics can cross the BBB. Nootropics are drugs that enhance learning and memory by facilitating the flow of information between the cerebral hemispheres. Nootropics also fortify the CNS against chemical and physical injuries. Nootropics are also used during the initial treatment stages of neurodegenerative diseases. Although this therapeutic group is structurally very diverse, the racetames subgroup is relatively structurally uniform. The racetames are characterised by the presence of a pyrrolidine-2-on moiety [5, 6]. Piracetam, 2-(2-oxopyrrolidine-1-yl)acetamide, is the most frequently prescribed drug of this group. Its more hydrophobic derivative nefiracetam, *N*-(2,6-dimethylphenyl)-2-(2-oxopyrrolidine-1-yl)-acetamide [7], is closely structurally related to the experimental drugs, the log BB values of which were estimated by means of regression models developed in this study (Figure 2).

## Results

### Development of regression models

The retention times of the initial set of 21 compounds (listed in Table 1) were measured; their log k′ values were also calculated. The log BB values were obtained from the literature [8–10]. A least squares analysis was used to measure the correlation between the log BB and log k′ values. The retention time values are the means of three repeated measurements.

This analysis, when correlated by a plain linear regression model in the general form (equation 3),

Eq. 3. $$\text{log}\;\text{BB}=\text{a}\;\text{log}\;{\text{k}}^{\prime }+\text{b},$$

led to poor correlation (equation 4):

Eq. 4. $$\begin{array}{l} \text{log}\;\text{BB}=0.331\;\;\text{log}\;{\text{k}}^{\prime }-0.148\\ \text{n}=21;\;\text{R}=0.447;\;\text{F}=4.740;\;\text{s}=0.452 \end{array}$$

Similarly, fitting to a simple quadratic model in the form (equation 5)

Eq. 5. $$\text{log}\;\text{BB}={\text{a}}_{1}{\left(\text{log}\;{\text{k}}^{\prime }\right)}^{2}+{\text{a}}_{2}\text{log}\;{\text{k}}^{\prime }+\text{b}$$

led to equation 6.

Eq. 6. $$\begin{array}{l} \text{log}\;\text{BB}=-0.301\;{\left(\text{log}\;{\text{k}}^{\prime }\right)}^{2}+0.461\;\;\text{log}\;{\text{k}}^{\prime }-0.035\\ \text{n}=21;\;\text{R}=0.486;\;\text{F}=2.781;\;\text{s}=0.454 \end{array}$$

Excluding the outliers improved the correlation. Two approaches to identify outliers were tested: (a) the stepwise exclusion of influential points, which were identified by statistical tests; (b) the stepwise exclusion of points with the greatest residual value. The residual value is the difference between a particular log BB value reported in the literature and the predicted value. The residual value is expressed as a percentage of the reported value. Approach (a) led to the exclusion of indomethacin, which was also identified as an outlier by both the Atkinson distance and its influence on the regression parameters. The linear model was then improved to the form of equation 7:

Eq. 7. $$\begin{array}{l} \text{log}\;\text{BB}=0.408\;\;\text{log}\;{\text{k}}^{\prime }-0.097\\ \text{n}=20;\;\text{R}=0.654;\;\text{F}=13.468;\;\text{s}=0.326 \end{array}$$

Indomethacin was also excluded as an outlier in the quadratic model. This model was thus improved to the form of equation 8:

Eq. 8. $$\begin{array}{l} \text{log}\;\text{BB}=-0.437\;\;{(\text{log}\;{\text{k}}^{\prime })}^{2}+0.601\;\;\text{log}\;{\text{k}}^{\prime }+0.070\\ \text{n}=20;\;\text{r}=0.733;\;\text{F}=9.856;\;\text{s}=0.302 \end{array}$$

Further improvement of both regression models was attained by excluding quinidine, a compound identified as an influential point by the Atkinson distance as well as Andrews-Pregibon statistics. The resulting linear model could be expressed using equation 9:

Eq. 9. $$\begin{array}{l} \text{log}\;\text{BB}=0.501\;\;\text{log}\;{\text{k}}^{\prime }-0.071\\ \text{n}=19;\;\text{R}=0.792;\;\text{F}=28.609;\;\text{s}=0.262 \end{array}$$

The corresponding quadratic model could be then written in the form of equation 10:

Eq. 10. $$\begin{array}{l} \text{log}\;\text{BB}=-0.303\;\;{(\text{log}\;{\text{k}}^{\prime })}^{2}+0.624\;\;\text{log}\;{\text{k}}^{\prime }+0.041\\ \text{n}=19;\;\text{R}=0.825;\;\text{F}=17.000;\;\text{s}=0.250 \end{array}$$

In models of both orders, salicylic acid was proposed as the additional outlier again by means of the Atkinson distance and Andrews-Pregibon statistics. The exclusion of salicylic acid led to a significant improvement in the first-order linear model (equation 11),

Eq. 11. $$\begin{array}{l} \text{log}\;\text{BB}=0.413\;\;\text{log}\;{\text{k}}^{\prime }-0.013\\ \text{n}=18;\;\text{R}=0.815;\;\text{F}=31.749;\;\text{s}=0.195 \end{array}$$

but not in the quadratic model. An insignificant increase in the linear correlation coefficient R led to a corresponding decrease in the Fisher-Snedecor test of model significance (F) value (equation 12):

Eq. 12. $$\begin{array}{l} \text{log}\;\text{BB}=-0.157\;{(\;\text{log}\;{\text{k}}^{\prime })}^{2}+0.484\;\;\text{log}\;{\text{k}}^{\prime }+0.041\\ \text{n}=18;\;\text{R}=0.829;\;\text{F}=16.498;\;\text{s}=0.194 \end{array}$$

Thus, the model expressed in equation 10 is the best quadratic model of our approach (a). In the linear model, only Andrews-Pregibon statistics identify codeine as an influential point. However, codeine’s exclusion led to a significant improvement of the model (equation 13):

Eq. 13. $$\begin{array}{l} \text{log}\;\text{BB}=0.405\;\;\text{log}\;{\text{k}}^{\prime }-0.036\\ \text{n}=17;\;\text{R}=0.855;\;\text{F}=40.596;\;\text{s}=0.169 \end{array}$$

The Atkinson distance, Andrews-Pregibon statistics, and the Y^2^ parameter each identified carbon disulphide as an influential point. Its exclusion led to the model expressed in equation 14:

Eq. 14. $$\begin{array}{l} \text{log}\;\text{BB}=0.379\;\;\text{log}\;{\text{k}}^{\prime }-0.054\\ \text{n}=16;\;\text{R}=0.883;\;\text{F}=49.601;\;\text{s}=0.141 \end{array}$$

This model was, again, markedly better than the previous one. The Atkinson distance and Andrews-Pregibon statistics now identified ibuprofen as an influential point. Its exclusion further improved the model (equation 15):

Eq. 15. $$\begin{array}{l} \text{log}\;\text{BB}=0.398\;\;\text{log}\;{\text{k}}^{\prime }-0.035\\ \text{n}=15;\;\text{R}=0.932;\;\text{F}=86.383;\;\text{s}=0.111 \end{array}$$

Diazepam, which had been further proposed to be an outlier due to its indicated Atkinson distance and influence to Y^2^, did not lead to a significant improvement of the model upon being excluded from the model. Notably, however, a negligible increase of R was accompanied by a commensurate decrease in the F value (equation 16):

Eq. 16. $$\begin{array}{l} \text{log}\;\text{BB}=0.373\;\;\text{log}\;{\text{k}}^{\prime }-0.045\\ \text{n}=14;\;\text{R}=0.934;\;\text{F}=82.624;\;\text{s}=0.101 \end{array}$$

Thus, equation 15 is the best linear model for approach (a).

The procedure for approach (b) started with the exclusion of caffeine, which had a residual value of 376.374 % of Y. The resulting model (equation 17) was only negligibly better than the previous model in terms of R and F values; the residual standard deviation s value was even greater in the new model (see equation 4):

Eq. 17. $$\begin{array}{l} \text{log}\;\text{BB}=0.346\;\;\text{log}\;{\text{k}}^{\prime }-0.162\\ \text{n}=20;\;\text{R}=0.458;\;\text{F}=4.766;\;\text{s}=0.461 \end{array}$$

The corresponding quadratic model (equation 6) identified phenytoin as the compound with the greatest residual value (366.911 % of Y). Its exclusion led to the model (equation 18)

Eq. 18. $$\begin{array}{l} \text{log}\;\text{BB}=-0.334\;{(\;\text{log}\;{\text{k}}^{\prime })}^{2}+0.478\;\;\text{log}\;{\text{k}}^{\prime }-0.015\\ \text{n}=20;\;\text{R}=0.491;\;\text{F}=2.699;\;\text{s}=0.465 \end{array}$$

This model was not better than the previous one (equation 6). These results indicated that exclusion of compounds as outliers based on their residual value is an unreliable method.

### Estimation of blood-brain permeation of novel racetames

**Fig. 3:**
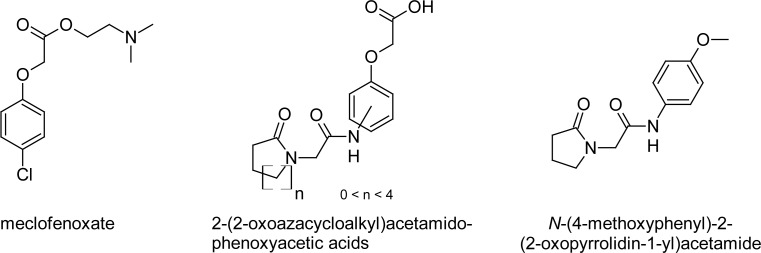
Structures of meclofenoxateand a series of novel 2-(2-oxoazacycloalkyl)-acetamidophenoxyacetic acids [11] and *N*-(4-methoxyphenyl)-2-(2-oxo-pyrrolidin-1yl)acetamide [7].

Final regression models, expressed by equation 10 for the quadratic model and by equation 15 for the linear model, were then used to estimate the ability of nine newly prepared [2-(azacycloalkyl)acetamido]phenoxyacetic acids [11] supplemented with *N*-(4-metoxyphenyl)-2-(2-oxopyrrolidin-1yl)acetamide [7] to cross the BBB (Figure 3). All compounds are potential cognitive enhancers and contain a ω-lactam moiety. These molecules are piracetam analogues and can also be classified in the 2-(azacycloalkyl)-acetamidophenoxyacetic acids series. This series shows some structural similarity to nootropic agents that are different than the racetames meclofenoxate and 4-chlorophenoxyacetic acid 2-(dimethylaminoethyl)ester (Figure 3). Compounds of this prediction set are shown in Table 2. The retention times (the means of three measurements), log k′ values, and the predicted log BB values are listed.

## Discussion

The two final regression models are, from a statistical viewpoint, equal to or better than previous models that have aimed to predict the structural properties that enable a molecule to permeate the BBB. The correlation of log BB and log k′ in the quadratic model (equation 10) is comparable to the model of Lombardo and co-workers [10], where log BB is dependent on the free energies of solvation. The plain linear model (equation 15) exhibits better characteristics, although it is based on a training set containing fewer members. Both models presented here also have better statistical parameters than those of Young and co-workers [3]. The models developed in this study are also comparable to more complex models that use computed parameters, including log BB dependence and descriptors expressing the electrotopological state of a molecule [12]. In conclusion, equations 10 and 15 confirm that the behaviour of a compound in a reversed-phase HPLC containing an embedded amide moiety can mimic the behaviour of the BBB. The models presume permeation by a passive diffusion mechanism. Notably, previous models are also based on this assumption [3, 10, 12].

Differences between the log k′ and log BB values of 2-(2-oxoazacycloalkyl)-acetamidophenoxyacetic acids are small. Notably, the values are predicted with both of the models in this study. Cruciani and co-workers [13] classified organic drugs and drug-like compounds by their predicted ability to cross the blood-brain barrier; these predictions were based on the log BB values of the structures. If log BB > 0, the compound readily crosses the BBB and reaches a greater concentration in the brain than in the blood. If 0 > log BB > 0.3, the compound may or may not cross the BBB. However, if log BB < −0.3, the compound is highly predicted not to cross the BBB. If this classification is applied to the results shown in Table 2, the members of the 2-(2-oxoazacycloalkyl)acetamido-phenoxyacetic acid series are predicted to not be able to permeate the BBB when the quadratic model is employed (equation 10). Using the plain linear model (equation 15) yields more optimistic results: FOK 27, 2-[2-(2-oxoazepane-1-yl)acetamido]phenoxyacetic acid, has a predicted log BB value of −0.244; FOK 26, 2-[2-(2-oxopiperidine-1-yl)acetamido]phenoxyacetic acid, has a predicted log BB value of −0.273; FOK 37, 3-[2-(2-oxoazepane-1-yl)acetamido]phenoxyacetic acetic acid, has a predicted log BB value of −0.281; and FOK 46, 4-[2-(2-oxopiperidine-1-yl)acetamido]phenoxyacetic acetic acid, has a predicted log BB value of −0.287. These predictions suggest that the molecules may possibly cross the BBB. In contrast, compound No. 12 from the patent of Betzing and co-workers [7], *N*-(4-methoxyphenyl)-2-(2-oxopyrrolidin-1yl)acetamide, has log BB values of 0.078 and −0.011 in the quadratic and plain linear models, respectively. Thus, compound No. 12 has a markedly stronger chance of permeating the BBB. Based on these results, the carboxy group seems to significantly limit a compound’s blood-brain permeability. This is in agreement with results of Platts and co-workers [8], who introduced a carboxylic acid indicator variable to obtain a multi-linear regression model for the dependence of log BB on several calculated structural properties. Some authors even demonstrated carrier-mediated transport mechanisms for both short- and long-chain aliphatic carboxylic acids as well as aromatic carboxylic acids [14]. The absorption of these experimental nootropics could be improved if members in the FOK series were transported through the BBB via such mechanisms.

## Conclusions

A reversed-phase HPLC that had been fitted with an embedded amide moiety was used to build two simple QSAR regression models between log BB and log k’, namely, a quadratic model as well as a plain linear one. These models were derived by a repeated linear regression that excluded outliers in a stepwise manner. The outliers were influential points. Both models showed that such an HPLC system can mimic the behaviour of the blood-brain barrier. In addition, estimating the log BB values of potentially novel racetam-type nootropics using these models, suggests that carboxy groups markedly inhibit a compound’s ability to pass through the BBB by a simple diffusion mechanism.

## Experimental

### Materials

All of the drug substances used in this study were of pharmacopoeial grade and were obtained and purchased from pharmaceutical suppliers. Potential nootropics, used for log BB estimation, were prepared in our laboratory as previously described [7, 11]. Their identities were confirmed by ^1^H-NMR, ^13^C-NMR, and IR spectroscopies. Compounds were at least 95% pure (HPLC). Methanol and water were of HPLC grade and were purchased from Merck, Darmstadt, Germany. Potassium dihydrogen phosphate, toluene, xylenes, propan-2-one, butan-2-one, and 1,1,2-trichloroethylene were all of analytical grade. Sodium hydroxide, sodium phosphate, and sodium azide, purchased from Lach-Ner, Neratovice, Czech Republic, were all of analytical grade. The reversed-phase analytical HPLC column with an embedded amide moiety, namely, the hexadecanoylamidopropyl substitution on silica gel hydroxyls, was purchased from Sigma-Aldrich, Prague, Czech Republic (Supelcosil ABZ Plus+, 150 × 4.6 mm, 3 μm particle size).

### HPLC equipment and measurement conditions and procedure

HPLC measurements were performed on a set consisting of an LCP 4020 isocratic pump (Ecom, Prague, Czech Republic), Rheodyne® (Rheodyne, Rohnert Park, USA). The system utilised a manual sample injector equipped with a 20-μL loop. The ABZ Plus+ column (above) was maintained at ambient temperature. An LCD 2083 UV-VIS detector was linked to Clarity integration software (Ecom, Prague, Czech Republic). A mobile phase consisting of methanol and a phosphate buffer (pH 7.4, 0.008 mol/L) was used. The flow rate was maintained at 0.6 mL/min. A wavelength of 254 nm was used for spectrophotometric detection. Solid compounds were dissolved in the mobile phase composition to a final concentration of 0.1 mg/mL. Aromatic liquids were diluted to 0.1 μL/mL; non-aromatic liquids were diluted to 1 μL/mL. Basic compounds, supplied as their salts, had been previously converted into their bases with sodium phosphate. Sodium azide at a final concentration of 0.1 mg/mL in the mobile phase composition, was used to determine the dead retention time; the determined value was 2.62 min.

### Data processing tools

Linear regression analyses were performed using QCExpert 2.5 Statistical Software (Trilobyte, Pardubice, Czech Republic) run on a Pentium 4 2.66 GHz computer.

## Figures and Tables

**Tab. 1.** Compounds of the training set and their parameters

**Compound name**	**log BB from literature**	**Retention time t_r_ [min]**	**Variance of t_r_**	**log k′**
propan-2-one	−0.15	3.430	5.492×10^−3^	−0.517
butan-2-one	−0.08	3.940	6.143×10^−3^	−0.303
1,1,2-trichloroethene	0.34	21.275	9.930×10^−3^	0.851
toluene	0.37	22.870	1.135×10^−1^	0.863
*o-*xylene	0.37	39.078	5.477×10^−1^	1.142
*p-*xylene	0.31	44.193	5.431×10^−1^	1.201
carbon disulphide	0.60	14.583	5.503×10^−4^	0.660
salicylic acid	−1.10	3.190	7.153×10^−3^	−0.672
acetylsalicylic acid	−0.50	3.095	6.836×10^−3^	−0.753
paracetamol	−0.31	3.610	1.213×10^−4^	−0.429
ibuprofen	−0.18	11.690	8.553×10^−2^	0.537
indomethacin	−1.26	16.316	1.399×10^−1^	0.716
phenazone	−0.097	4.085	1.965×10^−3^	−0.257
aminophenazone	0	4.845	3.190×10^−2^	−0.075
codeine	0.55	8.688	8.132×10^−3^	0.362
phenytoin	−0.04	9.600	2.430×10^−1^	0.423
caffeine	−0.055	3.812	5.557×10^−4^	−0.347
theophyline	−0.29	3.252	1.853×10^−4^	−0.626
quinidine	−0.46	34.427	1.517×10^−2^	1.082
physostigmine	0.079	8.812	2.385×10^−3^	0.371
diazepam	0.52	26.066	4.582×10^−1^	0.950

**Tab. 2.** Compounds of the prediction set with predicted log BB values.

**Cpd. code**	**Position of (2-oxoaza-cycloalkyl)-acetamido moiety at the benzene ring**	**n**	**t_r_**	**Variance of t_r_**	**log k**′	**Predicted log BB**	
						**Eq. 10 (quadratic)**	**Eq. 15 (linear)**
FOK 25	2	1	3.134	3.000×10^−6^	−0.717	−0.562	−0.320
FOK 35	3	1	3.138	1.000×10^−6^	−0.714	−0.559	−0.319
FOK 45	4	1	3.126	2.533×10^−5^	−0.725	−0.570	−0.323
FOK 26	2	2	3.293	2.533×10^−5^	−0.598	−0.440	−0.273
FOK 36	3	2	3.128	6.533×10^−5^	−0.723	−0.568	−0.322
FOK 46	4	2	3.156	3.333×10^−3^	−0.635	−0.477	−0.287
FOK 27	2	3	3.409	2.633×10^−5^	−0.528	−0.372	−0.244
FOK 37	3	3	3.262	1.233×10^−5^	−0.620	−0.462	−0.281
FOK 47	4	3	3.164	2.250×10^−6^	−0.693	−0.537	−0.310
Cpd. 12 [7]	-	-	5.656	2.633×10^−5^	0.061	0.078	−0.011

## References

[1] Norinder U, Haeberlein M (2002). Computational approaches to the prediction of the blood–brain distribution. Adv Drug Deliv Rev.

[2] Tamai I, Tsuji A (1996). Drug delivery through the blood–brain barrier. Adv Drug Deliv Rev.

[3] Young RC, Mitchell RC, Brown TH, Ganellin CR, Griffiths R, Jones M, Rana KK, Saunders D, Smith IR, Sore NE, Wilks TJ (1988). Development of a new physicochemicalmodel for brain penetration and its application to the design of centrally acting H2 receptor histamine antagonists. J Med Chem.

[4] Seiler P (1974). Interconversion of lipophilicities from hydrocarbon/water systems into octanol /water system. Eur J Med Chem.

[5] Gouliaev AH, Senning A (1994). Piracetam and other structurally related nootropics. Brain Res Rev.

[6] Malykh AG, Sadaie MR (2010). Piracetam and Piracetam-Like Drugs. From Basic Science to Novel Clinical Applications to CNS Disorders. Drugs.

[7] Betzing H, Biedermann J, Materne C (1980). Pyrrolidinones and pharmaceutical composition containing them. German Patent, DE.

[8] Platts JA, Abraham MH, Zhao YH, Hersey A, Ijaz L, Butina D (2001). Correlation and prediction of a large blood–brain distribution data set—an LFER study. Eur J Med Chem.

[9] Norinder U, Sjöberg P, Österberg T (1998). Theoretical Calculation and Prediction of Brain-Blood Partitioning of¨Organic Solutes Using MolSurf Parametrization and PLS Statistics. J Pharm Sci.

[10] Lombardo F, Blake JF, Curatolo WJ (1996). Computation of Brain-Blood Partitioning of Organic Solutes via Free Energy Calculations. J Med Chem.

[11] Farsa O, Dočkal M, Kováčiková J, Benešová M (2008). Synthesis of 2-{[2-(2-oxo-1-azacycloalkyl)acetamido]phenoxy}acetic acids and their activity as aminopeptidase M inhibitors. J Serb Chem Soc.

[12] Rose K, Hall LH (2002). Modeling Blood-Brain Barrier Partitioning Using the Electrotopological State. J Chem Inf Comput Sci.

[13] Cruciani G, Pastora M, Guba W (2000). VolSurf: a new tool for the pharmacokinetic optimization of lead compounds. Eur J Pharm Sci.

[14] Tsuji A (2005). Small Molecular Drug Transfer across the Blood-Brain Barrier via Carrier-Mediated Transport Systems. NeuroRx.

